# Supplementary material for: Advantages of Acute Brain Slices Prepared at Physiological Temperature in the Characterization of Synaptic Functions
Source: Front Cell Neurosci. 2020 Mar 19;14:63. doi: 10.3389/fncel.2020.00063 (PMC7096554; doi:10.3389/fncel.2020.00063)
Supplement: Supplementary file 1 [file Image_1.PDF]

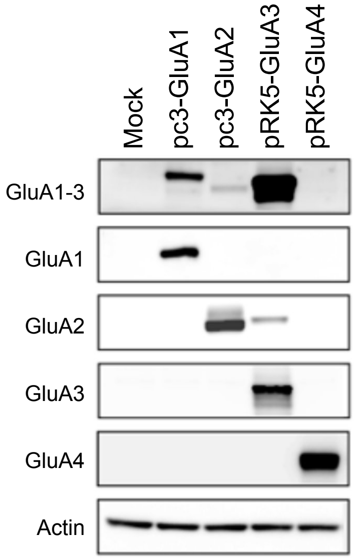

Figure S1. Specificity of anti-GluA1-3 antibody validated by Western blot analysis

COS-7 cells were transfected with expression vector for AMPA receptor subunits and analyzed by immunoblotting with anti-GluA1-3, anti-GluA1, anti-GluA2, anti-GluA3, anti-GluA4, or anti-actin antibody. The anti-GluA1-3 antibody reacted with GluA1, GluA2 and GluA3, but not with GluA4.

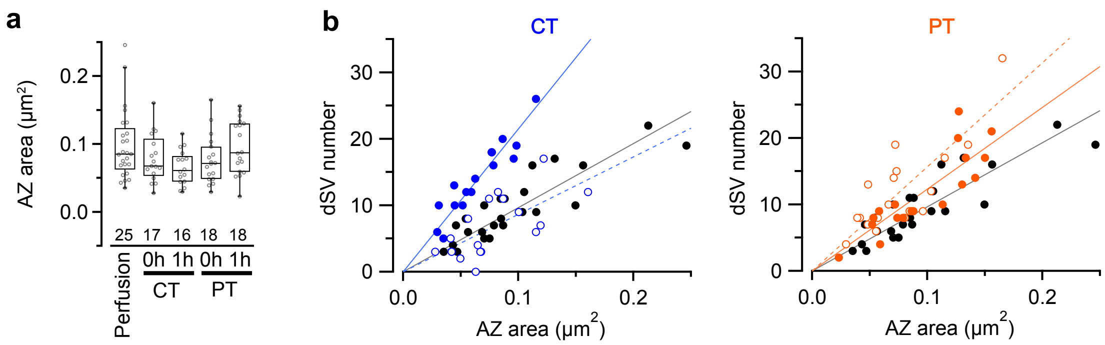

Figure S2. Correlation between AZ area and docked SV numbers

a, Summary of the AZ area in PF boutons. Numerals in plot indicate the numbers of analyzed boutons for each group.

b, Correlation between AZ area and dSV number in cold-cut (left) or warm-cut (right) slices. Black circles indicate the values in perfusion-fixed tissues. Open and closed circles indicate before and after 1-h recovery time, respectively. Lines show the regression lines of each group (blue: CT, red: PT, dotted: 0 h, solid: 1 h).
